# Supplementary material for: netSmooth: Network-smoothing based imputation for single cell RNA-seq
Source: F1000Res. 2018 Jul 10;7:8. Originally published 2018 Jan 3. [Version 3] doi: 10.12688/f1000research.13511.3 (PMC5814748; doi:10.12688/f1000research.13511.3)
Supplement: Supplementary file 1 [file f1000research-7-16868-s0000.tgz › 4da54602-f4d3-4c71-8727-372a734aa257.pdf]

Supplementary Figures S1-S14

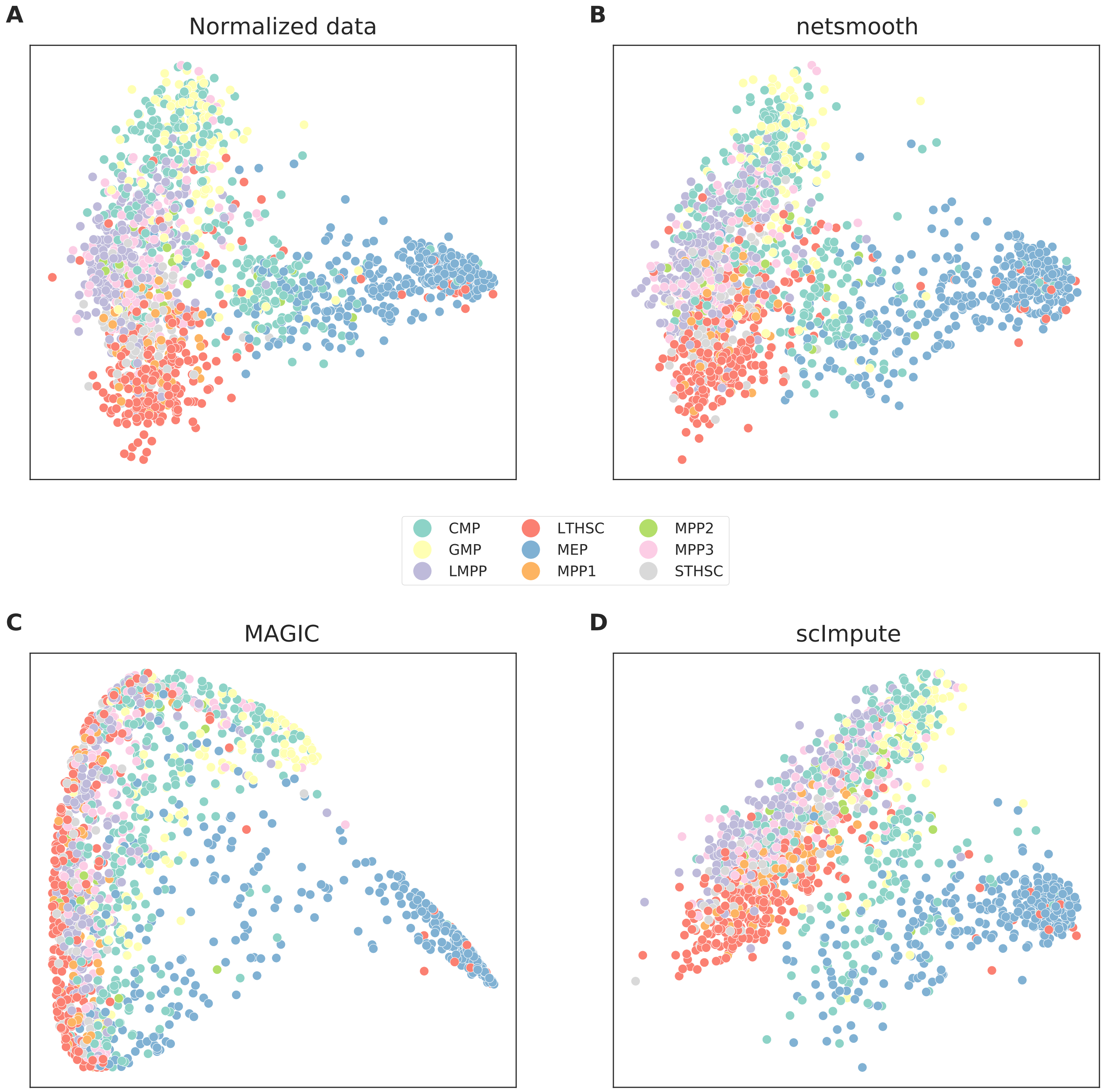

Figure S1. PCA plots of the HSPC dataset A) no preprocessing, B) after application of *netSmooth*, C), using scImpute, and D) after application of MAGIC.

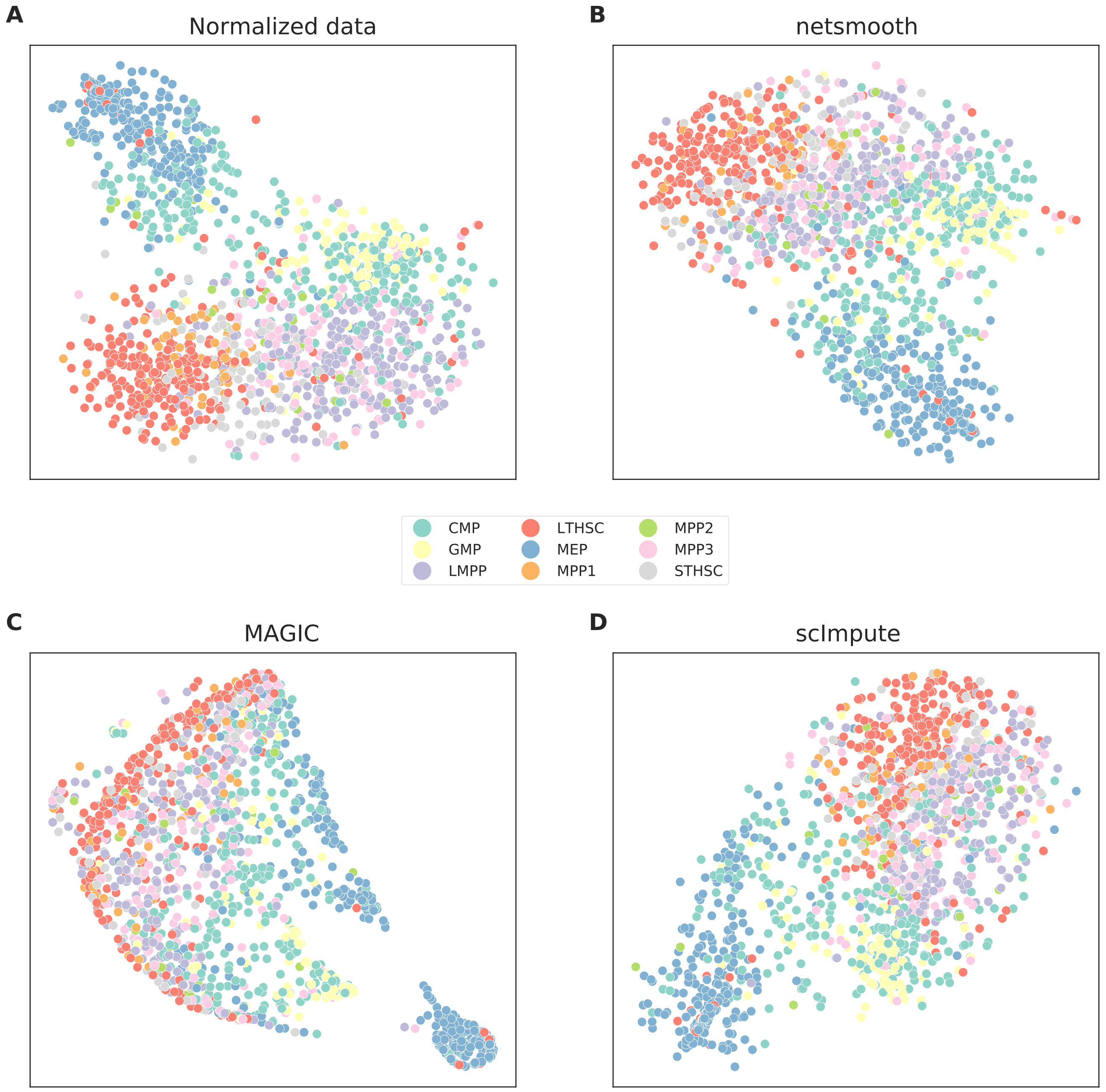

Figure S2. **t-SNE plots of the HSPC dataset A) no preprocessing, B) after application of *netSmooth*, C), using scImpute, and D) after application of MAGIC.**

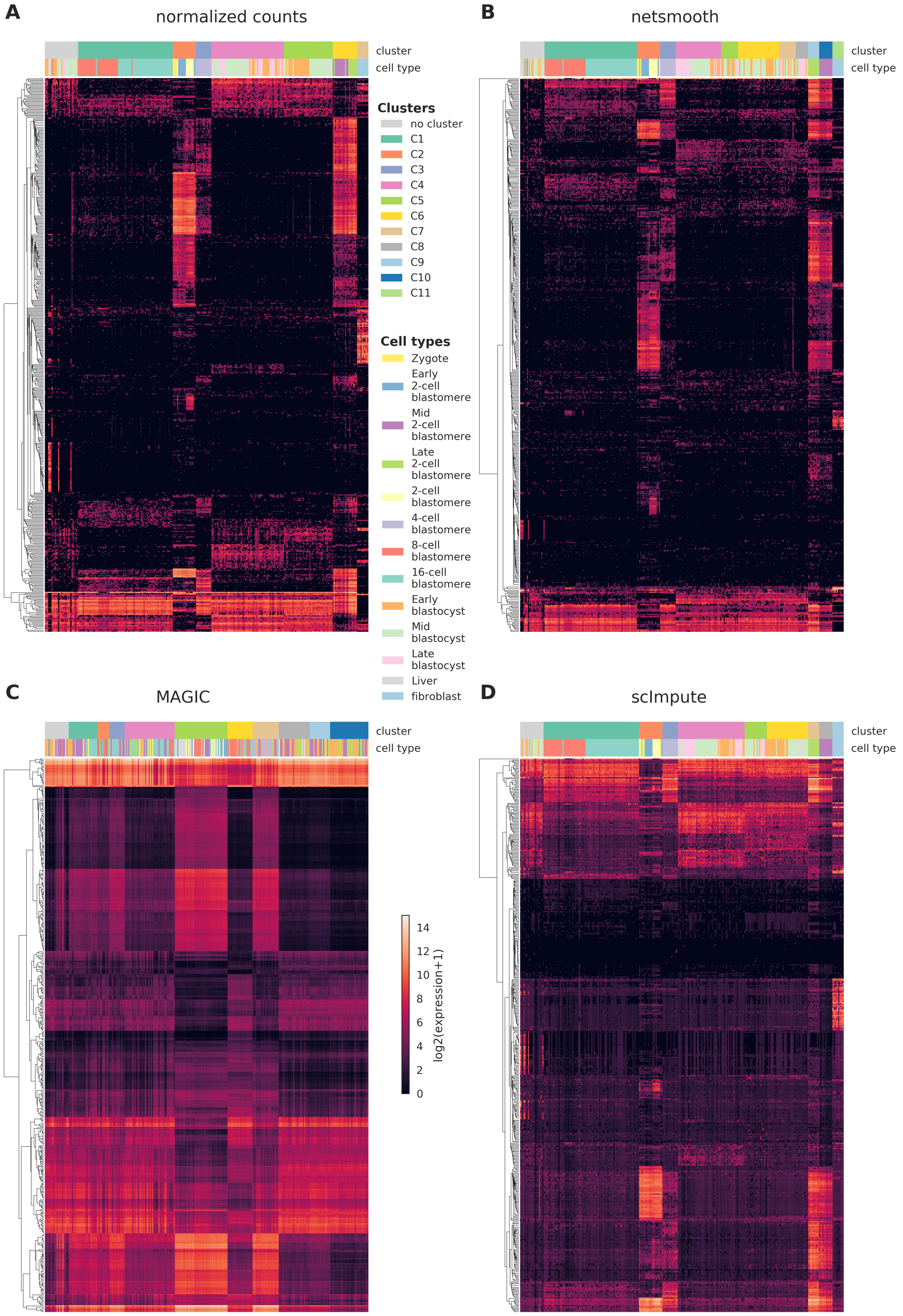

Figure S3. single cells from the embryonic development dataset were clustered using the robust clustering procedure, and the log-transformed expression values of the 500 most differentially expressed genes (by edgeR-QLF test adjusted P value) in any of the discovered clusters are shown in a heatmap, as well as cluster assignments and cell types. A) raw (no imputation), B) after application of *netSmooth*, C) missing values imputed using scImpute D) after application of MAGIC

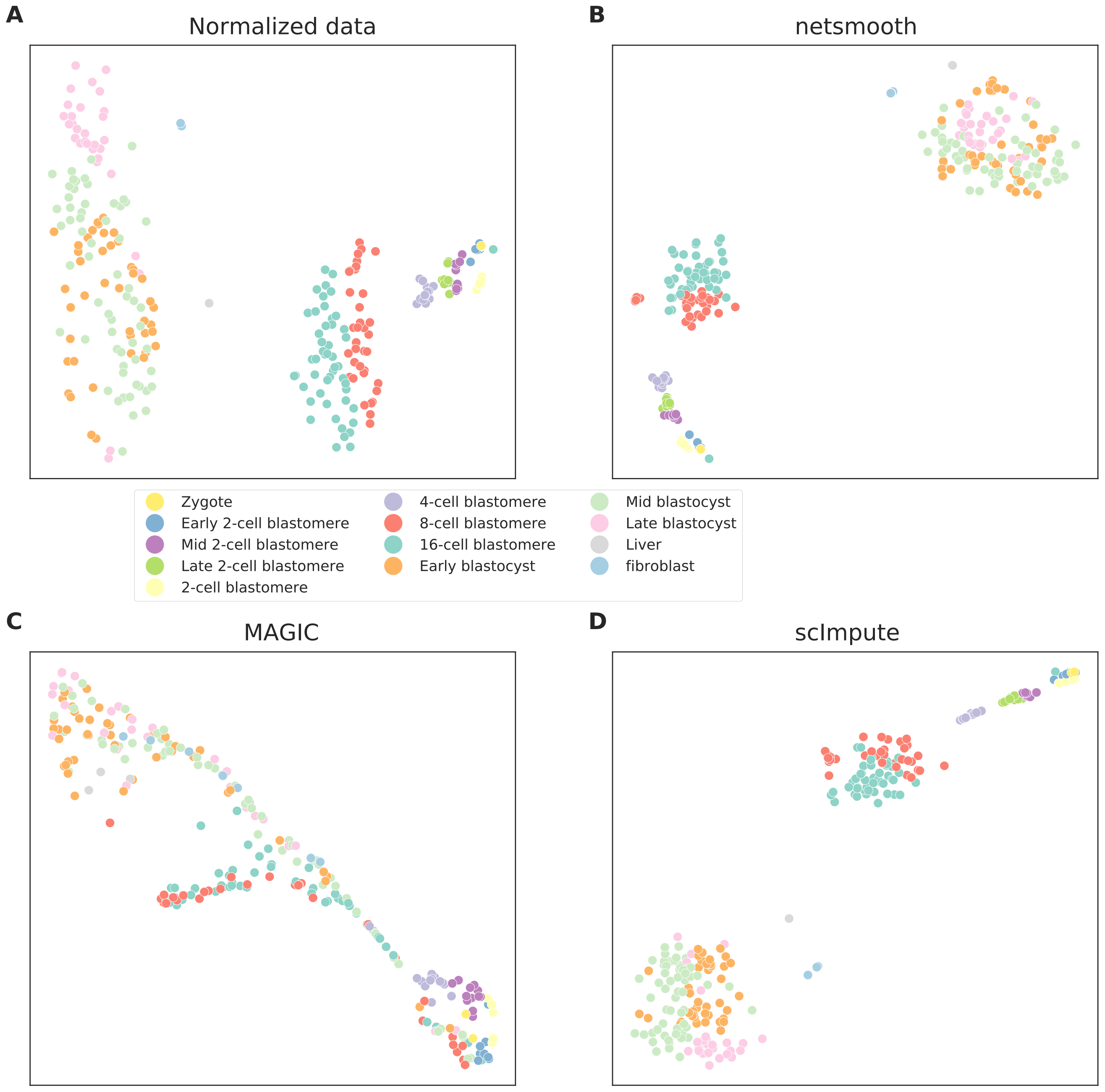

Figure S4. **t-SNE plots of the embvryonic development dataset A) no preprocessing, B) after application of *netSmooth*, C), using scImpute, and D) after application of MAGIC.**

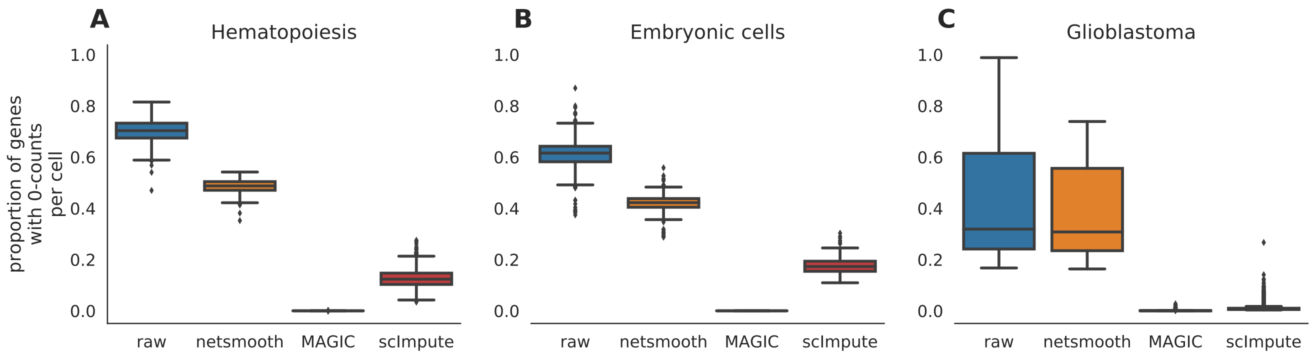

Figure S5. The proportion of genes with 0 counts is a proxy for technical dropouts. A) no preprocessing, B) after application of *netSmooth*, C), using scImpute, and D) after application of MAGIC.

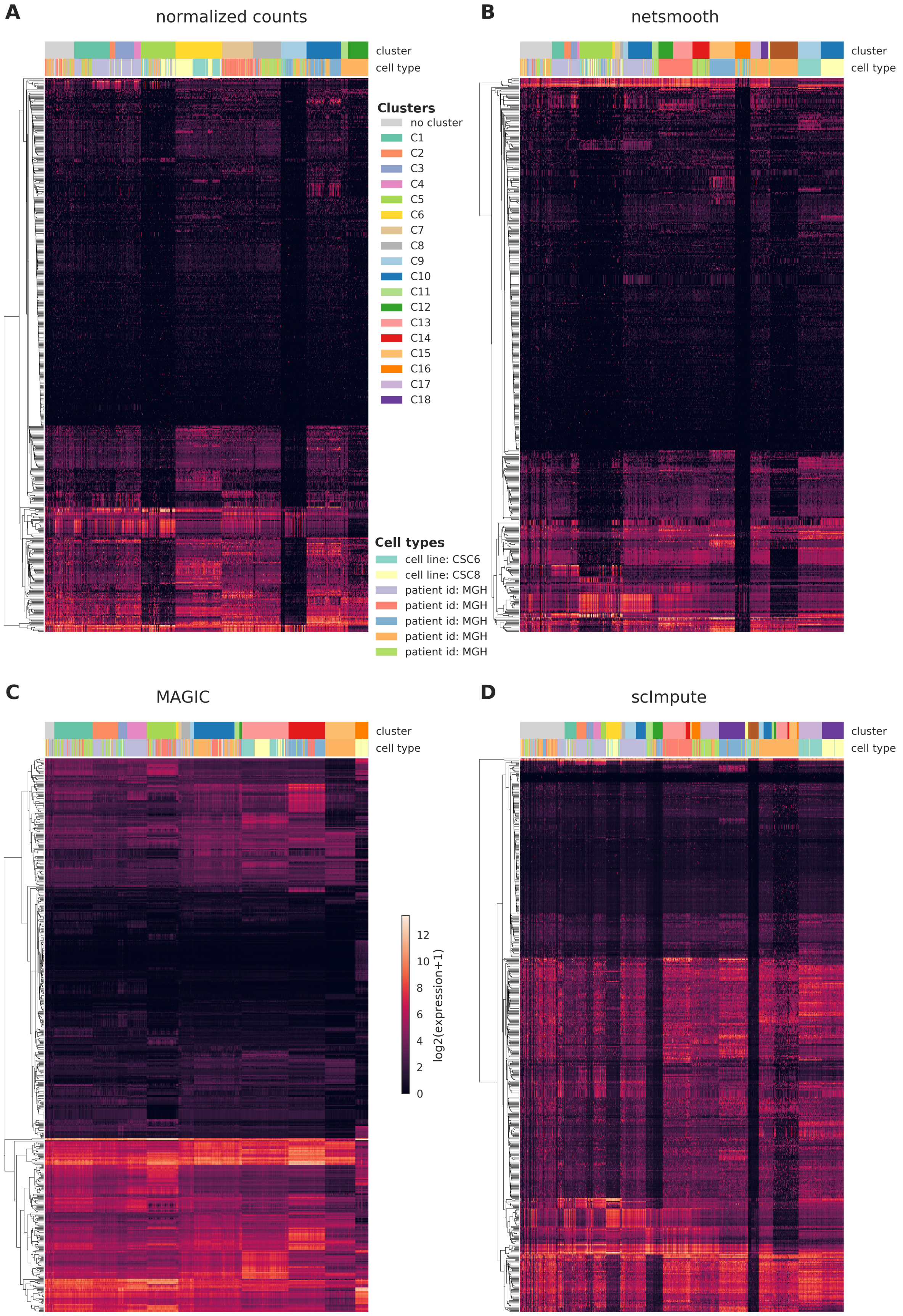

Figure S6. single cells from the glioblastoma dataset were clustered using the robust clustering procedure, and the log-transformed expression values of the 500 most differentially expressed genes (by edgeR-QLF test adjusted P value) in any of the discovered clusters are shown in a heatmap, as well as cluster assignments and cell types. A) raw (no imputation), B) after application of *netSmooth*, C) missing values imputed using scImpute D) after application of MAGIC

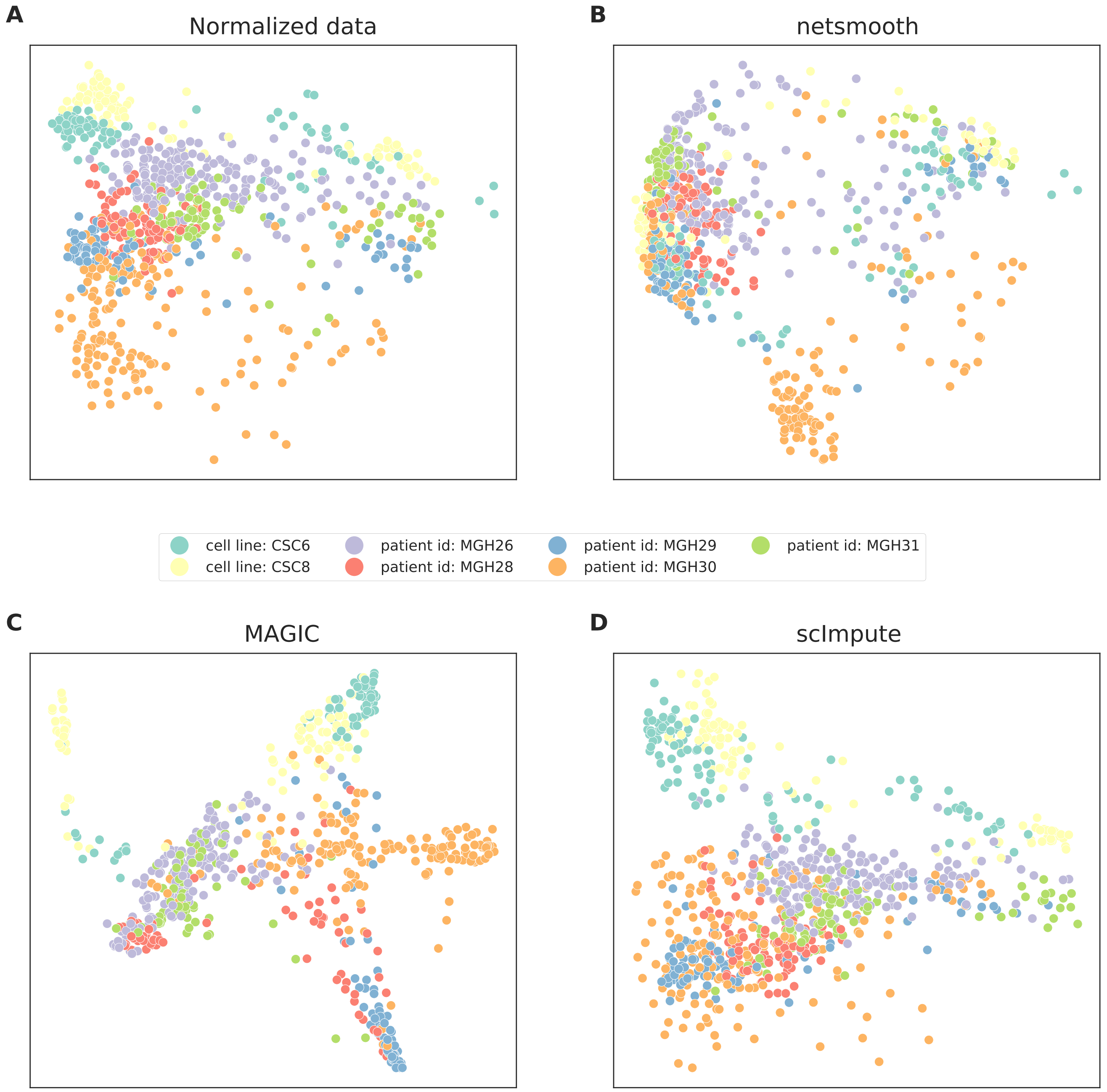

Figure S7. PCA plots of the glioblastoma dataset A) no preprocessing, B) after application of *netSmooth*, C), using scImpute, and D) after application of MAGIC.

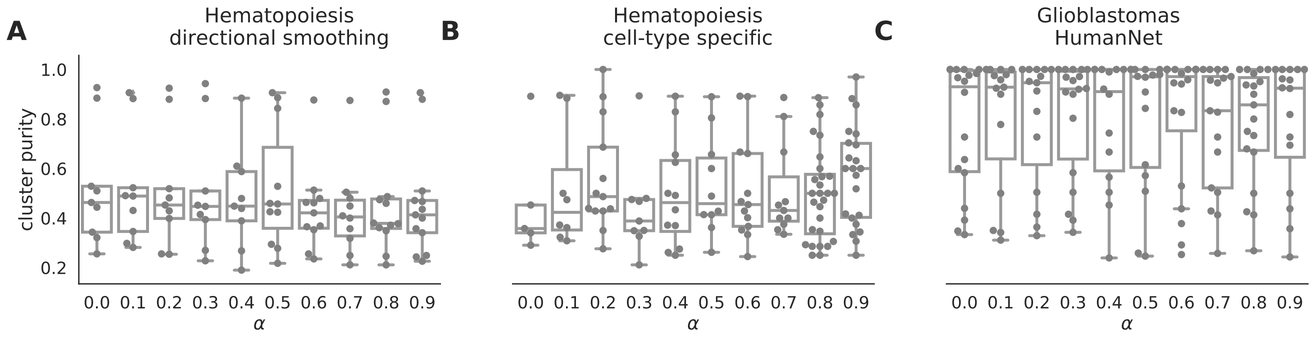

Figure S8. Cluter purity by smoothing parameter. A) for the hematopoiesis dataset with a directional (signed) graph, where inhibitory interactions have a negative edge weight. B) For the hematopoiesis dataset using a gene network with only genes that have a cell-type specific expression in any cell type. C) In the glioblastoma dataset using a gene network from HumanNet.

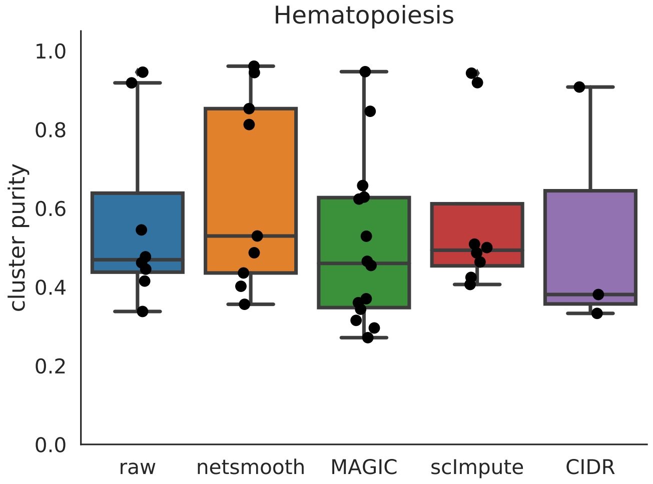

Figure S9. Cluster purity including CIDR. Same as Figure 3, with CIDR included.

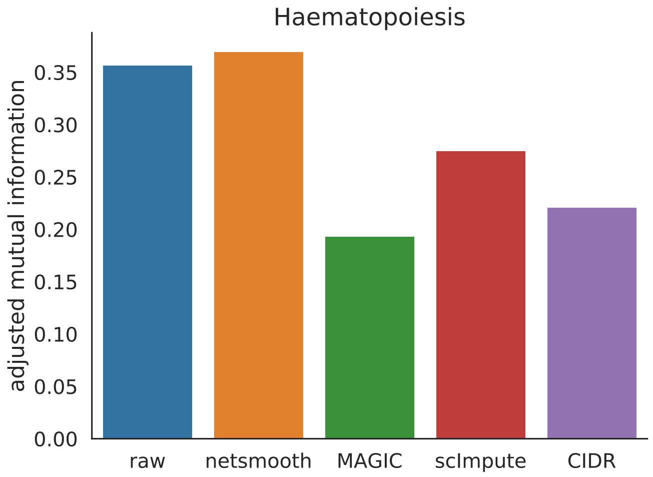

Figure S10. Adjusted mutual information including CIDR. Same as Figure 3, with CIDR included.

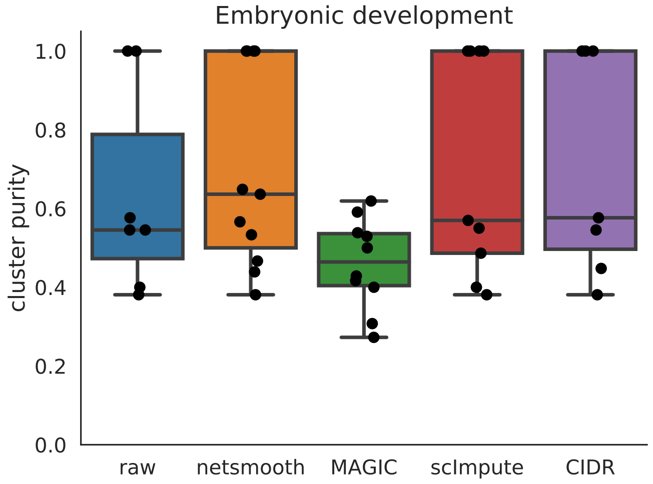

Figure S11. Cluster purity including CIDR. Same as Figure 5, with CIDR included.

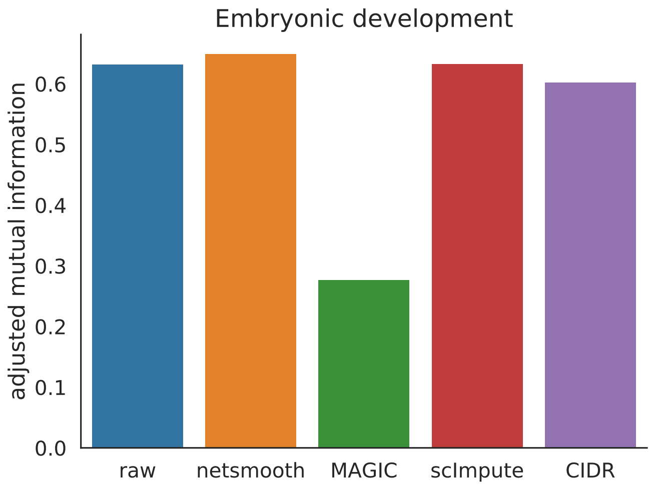

Figure S12. Adjusted mutual information including CIDR. Same as Figure 5, with CIDR included.

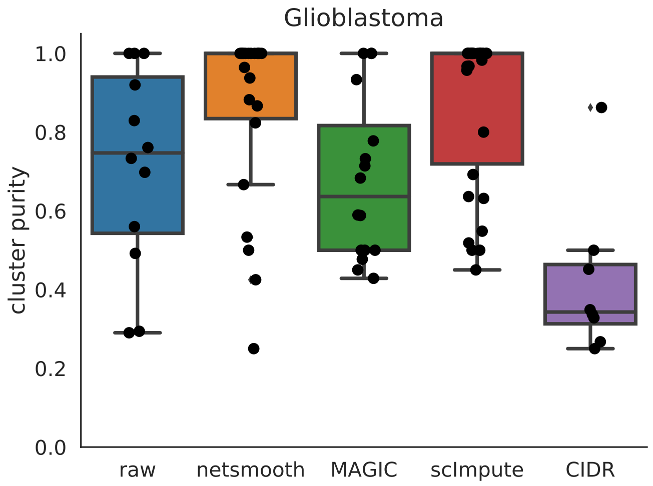

Figure S13. Cluster purity including CIDR. Same as Figure 7, with CIDR included.

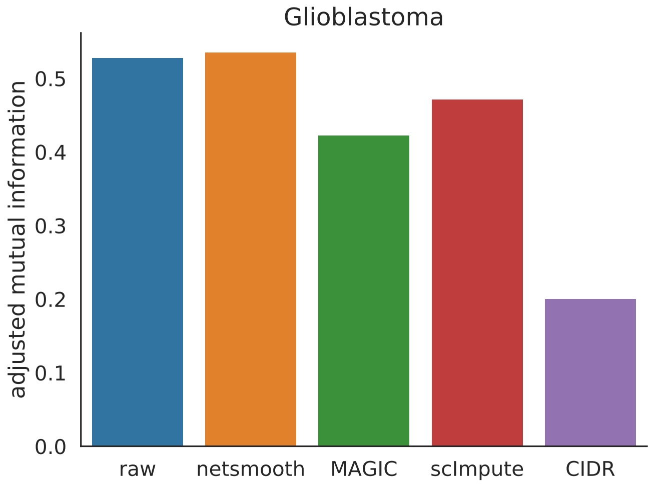

Figure S14. Adjusted mutual information including CIDR. Same as Figure 7, with CIDR included.
