# Supplementary material for: netSmooth: Network-smoothing based imputation for single cell RNA-seq
Source: F1000Res. 2018 Jul 10;7:8. Originally published 2018 Jan 3. [Version 3] doi: 10.12688/f1000research.13511.3 (PMC5814748; doi:10.12688/f1000research.13511.3)
Supplement: Peer review history

# Peer review: Referee response for version 2

**DOI:** 10.5256/f1000research.14981.r30156

**Published:** 2018-02-14

**Authors:** Siddharth Dey

**Affiliations:** 1 Center for Bioengineering, University of California, Santa Barbara, Santa Barbara, CA, 93106-5080, USA

In this manuscript, the authors have developed a new computational method to reduce technical biases that result from dropout events in single-cell mRNA sequencing experiments, a problem that particularly affects genes that are expressed at low levels. While single-cell mRNA sequencing has revolutionized our understanding of several biological systems in the last few years, the relatively low efficiency of amplifying small quantities of mRNA from a single cell results in dropout events that bias downstream analysis. To reduce this technical bias, the authors use information from protein-protein interaction maps to smoothen transcript counts across the entire dataset. Several groups are working on imputation based methods in single-cell mRNA-seq, and this manuscript presents an exciting approach to reduce technical noise. It would be helpful if the authors could clarify and discuss the points below in greater detail:

 1. Does the smoothening process bias against genes or gene networks that are not well represented in the protein-protein interaction network? One of the striking features of sc mRNA-seq is that it can identify the expression of specific genes that were previously not associated with a particular cell-type. Would this be impacted by netSmooth and can the authors provide examples from the datasets they have analyzed that netSmooth still retains these observations?

 2. There are several sc mRNA-seq methods (for example, CEL-Seq, Smart-Seq etc.) that are currently used by different labs. These methods have different features, such as, full-length transcripts or 3’ end sequencing, and the possibility of employing unique molecule identifiers. How do the 3 computational methods compared in this manuscript work on different experimental techniques?

 3. For most of the example datasets used in this manuscript, scImpute shows very similar performance to netSmooth on all 3 metrics used to compare the methods. Can the authors discuss how these two methods, while using different approaches, achieve similar performance. Are there conditions/datasets where one method would perform better than the other?

 4. The proportion of cells in robust clusters seems to be very sensitive to the choice of the free parameter in netSmooth (Figure 12). Further, in contrast to a statement in the text (last paragraph on page 10), the value of the free parameter that gives the highest proportion of cells in robust clusters does not correspond to the highest median cluster purity in the glioblastoma dataset. This high sensitivity to alpha can potentially pose a challenge. Can the authors comment on this? Could the authors propose alternate strategies for picking the optimal alpha value.

I have read this submission. I believe that I have an appropriate level of expertise to confirm that it is of an acceptable scientific standard.

# Peer review: Referee response for version 2

**DOI:** 10.5256/f1000research.14981.r30157

**Published:** 2018-02-05

**Authors:** Fernando J. Calero-Nieto, Fiona Kathryn Hamey

**Affiliations:** 1 Wellcome Trust and MRC Cambridge Stem Cell Institute & Cambridge Institute for Medical Research, Addenbrooke's Hospital, Cambridge, UK

In this manuscript, the authors describe a method for imputing values to overcome the problem of technical dropouts in single-cell RNA-seq datasets. As stated by the authors, the problem is well known and caused by technical limitations that affect low and high expressed genes. The approach discussed in the manuscript uses prior knowledge about protein interactions in order to smooth the expression values between pairs of genes encoding interacting proteins, thus reducing the number of zero values and altering the expression values of detected genes in each cell and influencing clustering and visualisation results.

 The proposed fundament is interesting and certainly worth exploring. However, there are a few considerations when using this type of approach: 1) the possible enhancement of known relationships to the detriment of the discovery of previously unknown ones; 2) Inferring dropouts using pre-known interactions could result in the overestimation of the expression of certain genes; 3) the gene relationships tend to be very cell-type specific so networks and PPIs should be different from cell type to cell type. In relation to the latter point, it is very interesting that the method is flexible enough to accept networks constructed from different sources.

 This manuscript compares the netSmooth algorithm to two existing approaches: Magic and scImpute. Overall, netSmooth presents an approach to smooth scRNA-seq data, which may prove useful in noisy datasets affected heavily by dropouts. However, there are several aspects of the manuscript that we found unclear or feel warrant further discussion.

  

- An important point is how the performance of these type of methods can be assessed. The authors decided to use a combination of clustered heatmaps, robustness and purity of the clustering, including a measurement of the correspondence between the 2 of them (AMI). Important downsides of this method are: a) external annotation of the datasets is required, which is not always available; b) robustness of the clustering seems to be strongly affected by the processing of the data, as the authors show in relation to MAGIC; c) the purity of the clustering could be strongly biased by the size of the clusters, since small clusters could have a greater chance to get a higher score. It would be interesting if the authors could comment on how their metrics are affected by the number of robust clusters identified. For example, could identifying more small clusters in the dataset have an effect in increasing the median cluster purity? And if so, is this a reliable measure for comparing between algorithms.
- It would be useful to include colour bars for the heatmaps. It should also be mentioned what scale the data is plotted using e.g. is it linear or log-transformed and is the scale comparable between all of the processed datasets? Additionally, some panels (for example 2C and 2D) have more clusters than there are colors shown in the cluster color key next to panel A, so the keys should be changed to match the data. 
- Figures showing the cluster purity are quite confusing. From the legend and methods, we understood that each point on the boxplot represents the purity for one of the clusters displayed in the clustered heatmaps. Yet in figure 2A, for example, there are 4 robust clusters found in the raw data, but 8 points for the raw clusters in figure 2B. It seems either that there is an error in one of the plots, or that we have misunderstood the cluster purity metric in which case it needs to be more clearly explained. 
- For continuity purposes, it would be better that either PCA or tSNE visualisations were shown for all dataset comparisons in the main figures instead of alternation between clustered heatmaps, PCA and tSNE. 
- In the introduction section, the authors mention the imputation programme CIDR along with scImpute and MAGIC, yet only compare the performance of netSmooth to scImpute and MAGIC. The authors should either include benchmarking against CIDR on the three datasets, or discuss why this is not appropriate. 
- When discussing applying netSmooth to the haematopoietic data, the authors state that “Figure 2a,b shows that after network-smoothing, we are able to identify clusters with a more pronounced differential expression profile. Further, many more of the genes identified as differentially expressed between the clusters (without smoothing) seem to have low and uninformative expression values overall.” However, from visual inspection of Figure 2 there appears to be very little difference in the expression levels of the differentially expressed genes in the two heatmaps, or in the number of genes with low expression levels. We are unsure exactly what the authors mean by “more pronounced differential expression profile” as it is hard to see a difference in the heatmaps. 
- The authors state that “Only MAGIC is able to increase the proportion of cells in this dataset which fall into robust clusters (Figure 3a), but only *netSmooth *leads to more biologically meaningful clusters, in terms of purity and AMI (Figures 3b,c), demonstrating that *netSmooth *can assist in cell type identification, and outperformed both MAGIC and scImpute in this task.” The increase in AMI in 3B is marginal compared to the raw data, and the proportion in robust clusters is higher for raw data than for netSmooth. There is also no clear improvement in the visualizations of figures S1 and S2 between netSmooth and raw data. Combined with the heatmaps in figure 2, we didn’t feel that there was compelling evidence that netSmooth was useful in cell type identification, and therefore this statement should be toned down. 
- In figure 4, it is hard to see how either netSmooth or scImpute offer an improved visualization compared to the raw data. This is backed up by very similar metric scores in Figure 5A and 5C between the raw, netSmooth and scImpute bars. Therefore the statement “Although MAGIC and scImpute reduce the 0-count genes further than *netSmooth *(Figure S1), they do not add as much clarity to the developmental stage signal inherent in the data.” appears to overstate how well netSmooth performs on this dataset in comparison to the other two algorithms. 
- In several places the text references the wrong supplementary figures. For example, in the sentence “Although MAGIC and scImpute reduce the 0-count genes further than *netSmooth *(Figure S1)” the authors appear to be actually referring to Figure S5. 
- In Figure S5, it should be clarified what is plotted. The legend needs to be changed to make it clear what this is showing. Is this the proportion of zero genes *per cell* in each dataset? Also, the data in this figure suggests that this method has a stronger effect on the expression of genes that are already expressed more than in the removal of zeros. The authors should comment on this in the main manuscript.
- When applying netSmooth to the tumor data, the authors assess the ability of their algorithm on the extent to which it separates cells from different samples, stating “it is also applicable in cancer, improving identification of tumor of origin for glioblastomas.” In fact, many researchers are actually interested in removing this effect in order to be able to compare similar cell types between different patients. Is it possible that netSmooth is actually enhancing “batch effect” in this dataset? It would be interesting to see whether netSmooth increases technical (rather than biological) batch effect in another dataset where a strong biological batch effect is not expected. 
- When assessing the importance of the PPI network structure, the authors calculate clustering metrics for randomly permuted networks. Can the authors comment on the fact some random networks have better cluster purity than the real network? Also, why do the authors not show AMI for these random clusters when it is often used to support the success of netSmooth compared to other approaches (e.g. in the haematopoiesis dataset)? 
- When discussing the parameter selection the authors state that “in the embryonic development dataset, we would choose alpha= 0.7 as the value that produces the highest cluster purity”. But in figure 11B it is actually alpha= 0.4 that has the highest cluster purity. It would be interesting if the authors commented in why there are at least 2 alpha values that get very similar maximum values for each dataset. Also, the figure would benefit from including alpha=0 values to compare with raw data.

We have read this submission. We believe that we have an appropriate level of expertise to confirm that it is of an acceptable scientific standard.
